# Multiple sclerosis-A disease on a dramatically rising trend in Iran: Review of possible reasons

**Published:** 2017-01-05

**Authors:** Mohammad Ali Sahraian, Mohammad Sahebkar, Rouhullah Dehghani, Milad Derakhshan-Jazari, Vahid Kazami-Moghaddam, Ebrahim Kouchaki

**Affiliations:** 1 Multiple Sclerosis Research Center, Neuroscience Institute, Tehran University of Medical Sciences, Tehran, Iran; 2 Department of Statistics and Epidemiology, School of Health, Sabzevar University of Medical Sciences, Sabzevar, Iran; 3 Social Determinants of Health Research Center, Department of Environment Health, School of Health, Kashan University of Medical Sciences, Kashan, Iran; 4 Department of Occupational Health, School of Public Health, Tehran University of Medical Sciences, Tehran, Iran; 5 Department of Environmental Health, School of Health, Neyshabur University of Medical Sciences, Neyshabur, Iran; 6 Department of Neurology, School of Medicine, Kashan University of Medical Sciences, Kashan, Iran

**Keywords:** Multiple Sclerosis, Causality, Prevalence, Incidence, Environmental Factors, Iran

## Abstract

There has been a global rising trend in recent years in the incidence of multiple sclerosis (MS). Despite being an MS low-risk region, this disease has also been recently on the rise in the Middle East. As part of the Middle East, Iran has not been spared either; however, the cause of this dramatic increase remains to be discovered. This study reviews possible reasons for this increase in Iran. Although many factors such as the increased rate of smoking, lifestyle changes, modernization, and contact with toxic solvents can be proposed as reasons for this sudden rise in the prevalence of MS in Iran, these factors cannot be taken as definite causes and further studies are required to prove their impact.

## Introduction

About 5-10% of the population in developed countries is affected by autoimmune diseases. These diseases are also a major cause of mortality and disability and increased medical costs.^1^^,^^2^ In terms of causality, autoimmune diseases can be created through a combination of various factors such as genetic, immunity, hormonal, and environmental factors.^3^^-^^5^ Multiple sclerosis (MS) is a neuronal infection in the central nervous system with a heterogeneous clinical and pathological composition that can develop suddenly and lead to death within only a few weeks or months. In many patients, it has a gradually increasing progress with a long and severe clinical course.^6^^,^^7^ Nevertheless, the progress of MS might cease or slow down after the first or second phase of its progress in benign cases.^8^

About 2 million people were estimated to have MS in 2003 across the world, about 400000 of whom were reported to live in the US. The majority of people with MS are in the 20-50 age range, and the disease tends to affect women more than men. Eastern Europeans are more affected by MS compared to Asians, Africans, and Latinos. This disease can cause death, disability, depression, physical impairment, and reduced quality of life. For instance, 50% of those affected require help with mobility and 10% need to use wheelchairs 15 years after the onset of the disease. In general, there are no treatments for MS.^9^^-^^12^ In addition to the absence of a treatment method, the cause of this disease is also still unknown. So far, various causes have been proposed by researchers for this disease, including infectious, environmental, and social factors. Many infectious diseases with various viral and bacterial causes have been investigated so far. Recent studies have been heavily focused on Epstein-Barr virus (EBV) infection. Acute EBV infection can remain in the body for life and its weaker form affects the B lymphocytes in 90% of the youth infected. In a number of case-control studies, EBV antibodies were noticed to have increased in the case group compared to the control group. In several well-designed studies, this virus was proposed as a risk factor for MS; however, in biological terms, this assumption remains only a possibility.^13^

Moreover, this disease is caused by various factors, including geographical latitude, vitamin D intake, skin color, immigration, meals, smoking, occupational contact with toxins and stress. The main factors involved in the development of this disease for which researchers have clear evidence include vitamin D, geographical latitude, and immigration.^3^^,^^14^ In the past, the pattern and distribution of MS were dependent on geographical latitude and was less prevalent in regions with higher latitudes. Overall, countries with a high prevalence of MS were mostly located in North America or Europe and countries closer to the equator boasted a lower prevalence of MS.^15^ Some current studies indicate that the global pattern of the prevalence of this disease has been changing. Some regions that used to be in the low-prevalence MS zone are now becoming moderate to high prevalence zones. The World Health Organization (WHO) published a report in 2008 on the global distribution of MS. 

**Figure 1:**
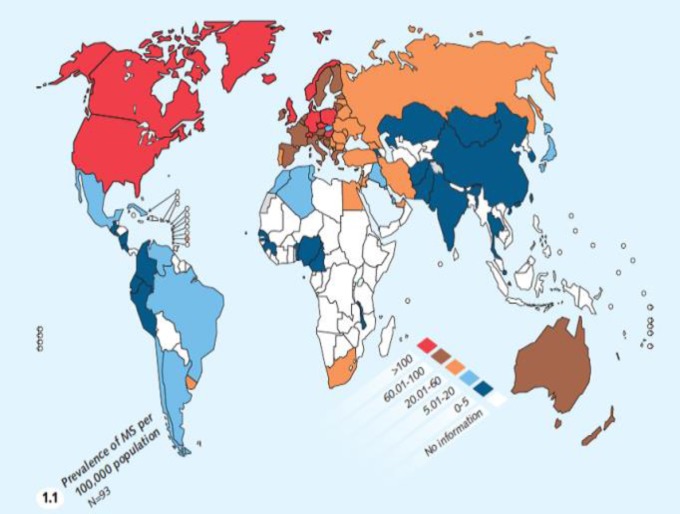
The global distribution of the prevalence of multiple sclerosis (MS) in 2008

According to figure 1, despite the prevalence gradient of the disease across the world, geographical distribution models previously proposed no longer apply to some regions such as the Middle East and particularly Iran.^16^^,^^17^ Although Iran is in the low-risk MS zone, studies indicate a dramatic rise in the prevalence of MS in Iran in recent years. This study was therefore conducted to investigate the possible reasons for the increase in the prevalence of MS in Iran. 

***MS status in the Middle East***

According to a study by Aljumah, et al., there are no proper epidemiological data in the Middle East about the prevalence, incidence or history of MS. Based on the Kurtzke Classification, the Middle East is located in a low-risk zone for MS. Nonetheless, recent studies argue that the prevalence of MS in the Middle East is increasing to moderate or high levels, and women are more affected by the disease.^18^

Other data available on MS and the Middle East have been presented in table 1 (given the full section to be presented on the status of MS in Iran, Iran has not been included in the list in this table).

According to table 1, there is an extensive time and place variation in the distribution of MS in the Middle East, and the geographical gradient previously detected in the region is changing.

***Status of MS in Iran***

Various studies conducted in different cities of Iran show a dramatic increase in the prevalence of MS in recent years.^29^^,^^30^ According to a study by Sahraian, et al., Iran has been switching from a low-prevalence zone for MS into a moderate to high prevalence zone. The results of the study show an estimated prevalence of about 52 per 100000 people in Tehran, Iran, 72.3% of whom are women and 27.7% men. The study also showed that the women to men ratio of the disease have been increasing from 2 in 2002 to 3.14 in 2007. The mean age of infection with MS is 27.24 years.^31^

In their study, Moghtaderi, et al.^32^ reported the prevalence and incidence of MS in southeast Iran as 13.96 per 100000 women and 2.67 per 100000 also reported a significant rise in the prevalence of men in 2010, with women to men ratio of 2.18, and MS in Iran compared to previous years.

**Table 1:** The prevalence of multiple sclerosis (MS) in countries in the Middle East

**Authors** **(references)**	**Country**	**Prevalence of MS**
Inshasi and Thakre^19^	Emirates	54.77 per 100000 people in 2007
Al-Hashel, et al.^20^	Kuwait	14.77 per 100000 people in 2000
Bohlega, et al.^21^	Saudi Arabia	40 per 100000 people in 2008
Al-Araji and Mohammed^22^	Iraq	300 patients diagnosed in the country in 2000
Yamout, et al.^23^	Lebanon	Estimated 1200-1800 patients in the country in 2008
El-Salem, et al.^24^	Jordan	39 per 100000 people in Amman
Radhakrishnan, et al.^25^	Libya	5.9 per 100000 people in 1982-1984
Tharakan, et al.^26^	Oman	4 per 100000 people in 1990 to 2000
Attia Romdhane, et al.^27^	Tunisia	12 per 100000 people in 1985
Dehghani, et al.^28^	Iran	44.53 per 100000 people in 2011

**Figure 2:**
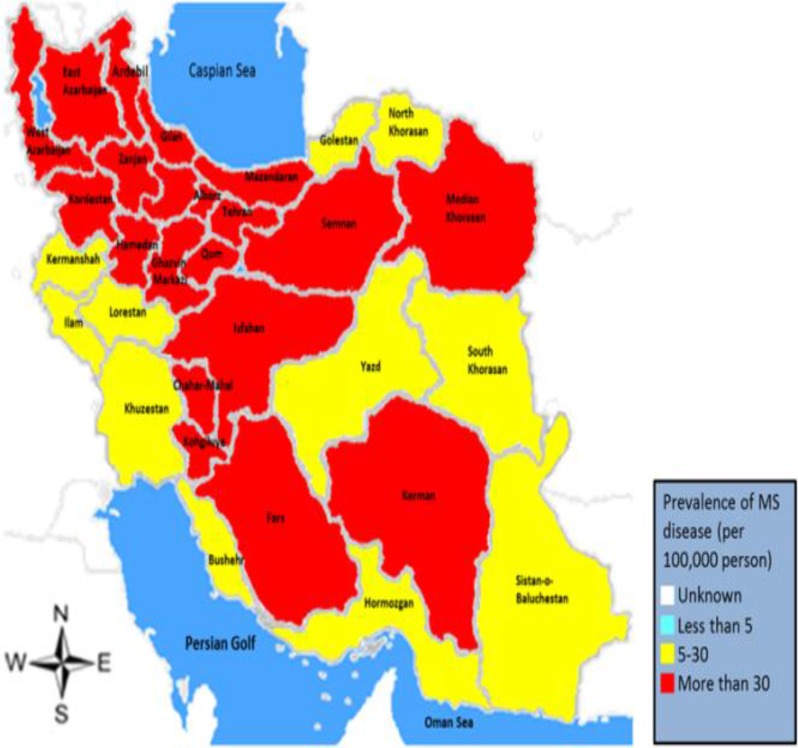
Status of multiple sclerosis (MS) in different provinces in 2011

The dramatic rise in the prevalence of MS was also noticed in Isfahan, Iran, in a study conducted by Etemadifar and Abtahi.^33^ Recent reports have proposed Isfahan as a city with the highest risk of MS in Asia and Oceania. The authors also suggested the design and implementation of control and screening programs for the prevention of this indiscriminately increasing prevalence. An ecological study conducted by Dehghani, et al.^28^ also revealed the dramatically growing trend of MS throughout Iran, with a prevalence increasing from 26.24 per 100000 people in 2006 to 44.53 per 100000 in 2011, and of the total of 31 provinces, 19 showed a moderate prevalence (5 to 30 patients per 100000 people) and 8 had a high prevalence (more than 30 patients per 100000 people), and only 3 had a low prevalence (< 5 patients per 100000 people); meanwhile, in 2011, only 11 provinces showed a moderate prevalence and the rest showed a high prevalence (Figure 2).

***Lifestyle Changes associated with Modernization, Industrialization, and Urbanization***

According to a WHO report, the use of tobacco and diets rich in fat, salt and sugar, which can lead to hypertension and obesity, and the increased use of packaged foods along with a sedentary lifestyle, might have emerged more as consequences of industrialization, urbanization, economic growth and globalization and can contribute to the development of chronic diseases.^34^ Based on a national census in Iran, there has been a tremendous rise in the rate of urbanization over the past few decades.^35^ A study by Ghassemi, et al. confirmed the rapidly changing nutrition pattern in Iran as well as the changing pattern of mortality and birth rates. The tendency toward urbanization was also shown to have occurred speedily among the Iranian population due to the unstable socioeconomic conditions. Furthermore, a poor nutrition pattern has taken over the entire Iranian population and overeating has become an integral part of life in one-third of the population. All these factors can predispose the individual to a variety of diseases. In the female population of Iran, obesity is a serious risk factor, which may be associated with many chronic diseases.^36^ In a study conducted in the US on 8983 patients with MS, 25.0% were obese and 31.3% were overweight, and 18.2% were exposed to the risk of alcohol abuse either through themselves or their relatives.^37^

In addition to the change in nutrition patterns, the quality of food items has also been dramatically changing. Some individuals and industries commit food frauds for making greater profits, which may lead to people’s deprivation of good food or the intake of harmful food products, causing various diseases. Food fraud was an existing crime in Iran as confirmed by Dehghani, et al.^38^

***Reduced vitamin D intake***

Many studies have proposed vitamin D as the key factor in the prevention of MS.^39^ Vitamin D deficiency is also the cause of many other chronic diseases aside from MS.^40^ A cohort study conducted on 95310 women from 1991 to 2002 conducted a regular investigation of the subjects’ vitamin D intake and their other dietary features using a validated nutrition questionnaire. Of the entire study population, 173 women developed MS in the course of the study and vitamin D intake was found to be inversely related to the risk of developing MS.^41^ Studies indicate that vitamin D deficiency is an epidemic condition in 20-25% of the population in the US, Canada, Europe, Asia, and Australia.^42^ Considering the reduced physical activity levels in countries of the Middle East as a result of lifestyle changes caused by the rise of industrialization and urbanization, sun exposure has been decreasing in this region (according to a WHO report). The lower exposure to the sun can be a factor in this region reduced rate of daily sunlight intake,^43^ which can significantly increase the development of MS in residents of this region and subsequently of Iran.^44^ It should be noted that although vitamin D is most likely the best dietary composition for the prevention of MS, other nutritional factors and even lifestyle-related tainting factors, too, can have a decisive role. Tainting factors may include other vitamin sources aside from vitamin D since vitamin D is normally absorbed in the presence of other vitamins.^41^

***Economic growth and living standards***

**Figure 3:**
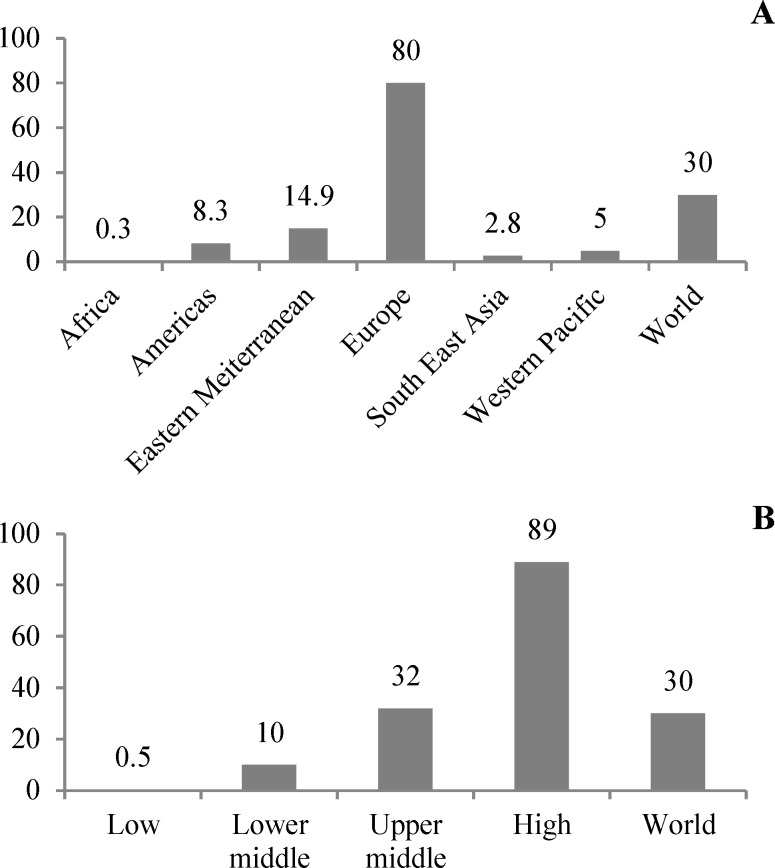
The prevalence of multiple sclerosis (MS) in different continents and by income level in different countries

The increasing rate of urbanization and economic growth in recent years in the Middle East may be involved in the improved lifestyle of the residents of this region.^28^ Some studies suggest a greater risk of developing MS in individuals with higher living standards so that the immune system’s adaptation to foreign agents is poorer in individuals with a better economic status during childhood, which can itself be a factor for the increased risk of the development of MS.^3^ According to a report published by the WHO, higher income countries were shown to have a higher prevalence of MS compared to poorer countries (Figure 3).

The factors involved in the countrywide increasing trend of the prevalence of MS have been listed and explained.

Figure 3 presents two diagrams showing the prevalence of MS in different continents and by income levels in different countries.^17^ It should also be noted that there is a poorer access to diagnostic facilities in the less developed countries compared to the developed countries, which might be a cause for underestimation in the reports. These differences are large enough to somewhat dismiss the poorer access to diagnostic facilities.

A: Prevalence of MS per 100,000 population in WHO regions and the word (N-93); B: Prevalence of MS per 100,000 population in different income groups of countries (N-93)

***Smoking***

Smoking is a major cause of many different chronic diseases.^45^ Many studies have associated smoking with the development or the increased risk of the development of MS.^46^ A study conducted in Norway showed that the risk of developing MS was 1.81 times greater in smokers than in those who have never smoked.^47^ Smoking increases the frequency and duration of respiratory infections and might, therefore, cause the recurrence of MS. However, there is a gap in reports on the relationship between MS and smoking or problems associated with pathological diseases. For instance, the results of a study conducted in Isfahan suggested a significant increase in the rate of the development of MS in women compared to men in recent decades; however, only 1.4% of the population of women were smokers in Iran, which does not suggest a significant relationship between smoking and MS.^48^ The absence of such relationship can be attributed to the different effects of smoking on the immune system. One of the major factors contributing to the development of chronic diseases is passive or second-hand smoking.^49^^,^^50^ The rate of smoking has been dramatically increasing in Iran; according to reports, about 60 billion cigarettes are smoked in Iran every year. An investigation conducted by the Iranian Ministry of Health confirms the growing trend of smoking cigarettes in recent years.^51^^,^^52^ A study conducted by Dehghani, et al.^28^ on the relationship between the prevalence of MS and lifestyle showed the greater prevalence of MS in provinces with a larger number of male smokers; however, this study was ecological and further studies are required to better demonstrate the impact of smoking.

***Air pollution***

Another possible factor for the development of MS is the greater air pollution in urban areas. The role of this factor is becoming more pronounced by the day, as studies have shown that increased urbanization leads to increased air pollution. Investigations have shown that air pollution (especially with particles such as PM10) can increase the risk of the development of MS.^53^^-^^55^ In recent years, air pollution (especially with PM10) has become a cause for great concern in many areas of Iran.^56^^,^^57^ For instance, in a study conducted in 2011 in Kashan, located in the high MS prevalence province of Isfahan, Dehghani, et al.^58^ found that the city’s air quality was acceptable only in 177 days of the year and that particulate matters were the main cause of air pollution in this city.^59^ The adverse effects of this factor require further investigations, especially in developing countries.

***Radon***

The majority of studies conducted to date on the effect of radiation absorption have been concerned with ultraviolet rays and their protective effects against MS. However, a limited number of studies state the lesser considered hypothesis that radon can be a potential risk factor for the development of MS.^60^ Radon is naturally emitted from the soil in some areas of Iran such as Ramsar, Iran. Some rocks and stones can naturally absorb the emitted rays, including decorative stones, especially granites, which tend to be commonly used in buildings in Iran without any supervision or prior evaluation.^61^^,^^62^ Although this element is only proposed as a potential risk factor for the development of MS, further investigations are required to fully understand the particular conditions in Iran.

***Occupational and Nonoccupational Contact with Chemicals***

Studies conducted in various parts of the world consider contact with industrial solvents as a factor contributing to the development of MS. However, the evidence seems insufficient, and this hypothesis has not yet been proven.^63^^-^^65^ No proper studies have been conducted in Iran on the link between contact with chemicals or special industrial solvents and MS. Nevertheless, according to some studies, contact with chemicals could entail a high risk for the development of this disease.^66^^,^^67^ For instance, Dehghani, et al.^68^ suggested that toxic chemicals are too easily available to people and emphasized the lack of specific rules and regulations for the purchase of these substances in Iran, which can increase the likelihood of developing certain chronic diseases, such as MS, which can be caused by contact with chemicals.^69^^,^^70^

## Conclusion

This study reviewed factors that can increase the prevalence of MS in Iran. The results obtained suggest a close link in Iran between MS and lifestyle changes, modernization, industrial growth, and urbanization. In recent years, the risk of developing diseases such as MS has been increasing in urban areas of Iran due to the changes in lifestyle, the increase in the urban population and the subsequent increase in air pollution. These factors have only been suggested as potentially effective, and future controlled studies could help further examine the relationship between MS and these factors.

## Notes:

**How to cite this article:** Sahraian MA, Sahebkar M, Dehghani R, Derakhshan-Jazari M, Kazami-Moghaddam V, Kouchaki E. Multiple sclerosis-A disease on a dramatically rising trend in Iran: Review of possible reasons. Iran J Neurol 2017; 16(1): 34-40.
